# On the pragmatic and epistemic virtues of inference to the best explanation

**DOI:** 10.1007/s11229-021-03338-7

**Published:** 2021-08-29

**Authors:** Richard Pettigrew

**Affiliations:** Department of Philosophy, University of Bristol, Bristol, UK

**Keywords:** Bayesianism, Inference to the best explanation, Evidence, Credences, Dutch Book argument, Dutch Strategy argument, Accuracy, Pragmatic arguments, Abduction

## Abstract

In a series of papers over the past twenty years, and in a new book, Igor Douven (sometimes in collaboration with Sylvia Wenmackers) has argued that Bayesians are too quick to reject versions of inference to the best explanation that cannot be accommodated within their framework. In this paper, I survey their worries and attempt to answer them using a series of pragmatic and purely epistemic arguments that I take to show that Bayes’ Rule really is the only rational way to respond to your evidence.

Once, when we were young, my friend Robert thought we saw a ghost. We were at his house one evening. As we walked along the hallway to the kitchen to make some snacks, we passed the bathroom and we saw that it was empty. As we chatted in the kitchen, we saw a shadow pass the kitchen door on the way to the bathroom. His father’s, surely. Then we saw the same shadow pass back again in the other direction, on the way from the bathroom. And then it happened. We saw a second shadow leaving the bathroom. But how could that be? The bathroom was empty when we passed it. We’d seen one shadow pass and return. There was only one route to the bathroom and it passed the kitchen door that we were facing. So whose was the second shadow? I maintained we must have been distracted when his mother’s shadow passed to go to the bathroom after his father, and the second shadow was hers returning in the other direction. But Robert concluded it must be a ghost, and he believes that to this day.

It’s natural to say that both Robert and I used inference to the best explanation first to arrive at and then to justify our different conclusions. We shared the same evidence: we’d both seen the bathroom empty; we both knew the layout of the house; we’d both seen the first shadow entering and then leaving the bathroom; we’d both seen the second shadow leaving the bathroom. I thought the best explanation was that the shadow belonged to Robert’s mother, and we’d simply missed her passing to go to the bathroom; Robert favoured an explanation that posited a ghost.

As the name suggests, philosophers think of inference to the best explanation as a rule of inference. Indeed, it is often listed as one of the three species of inference: deduction, induction, and inference to the best explanation, also known as abduction (Lipton 2004; Douven 2017, 2021).

**Inference to the best explanation (rule of inference)**

From

1. *E*; and
2. *H* is the best explanation of *E*;

infer

(C) *H*.

As such, it gives rise to a norm that governs our beliefs:

**Inference to the best explanation (norm for beliefs)**

You should believe the best explanation of your total evidence.

But we can also think of inference to the best explanation as a norm that governs how we change our degrees of belief or credences when we receive new evidence:

**Inference to the best explanation (norm for credences)**

You should be more confident in better explanations of your total evidence than in poorer ones.^1^

So, if $H_1$ is a better explanation of *E* than $H_2$, and if *p* is our prior credence function and $p_E$ is our posterior credence function after learning *E*, then $p_E(H_1)$ should be higher than $p_E(H_2)$.

Now of course there are other norms we take to govern our credences, and they include norms that govern how to set our posteriors given our priors and our evidence. So we might worry that the explanationist norms just sketched will conflict with them. The norms I have in mind are the Bayesian ones. Here’s the first—it’s a synchronic norm that governs your credences at any time.

**Probabilism** Your credences at any given time should satisfy the probability axioms.

That is, if your credence function *p* is defined on a finite algebra of propositions ${\mathcal {F}}$, as we’ll assume throughout this paper, then

(i) $0 \le p(X) \le 1$ for all propositions *X* in ${\mathcal {F}}$;
(ii) $p(\bot ) = 0$ and $p(\top ) = 1$, whenever $\bot$ is a contradiction and $\top$ a tautology; and
(iii) $p(X \vee Y) = p(X) + p(Y) - p(XY)$ for all *X*, *Y* in ${\mathcal {F}}$.

**Bayes’ Rule** When you receive new evidence, you should update your credences by conditioning your prior credences on your total evidence at that point, providing your prior assigns at least some credence to that total evidence; if it doesn’t, you can update in any way you please.

That is, if *p* is your prior, and $p_E$ is your posterior when your total evidence is *E*, and $p(E) > 0$, then it ought to be that

$$\begin{aligned} p_E(X) = p(X|E) := \frac{p(XE)}{p(E)} \end{aligned}$$

Now, it is common to point out that Bayes’ Theorem allows us to write Bayes’ Rule in a couple of more useful ways:

**Bayes’ Rule (combined with Bayes’ Theorem)** If $p(E)> 0$, it ought to be that

$$\begin{aligned} p_E(X) = p(X|E) = \frac{p(E|X)p(X)}{p(E|X)p(X) + p(E |\overline{X})p(\overline{X})} \end{aligned}$$

And, more generally, if $H_1, \ldots , H_n$ is a set of mutually exclusive and exhaustive hypotheses, then if $p(E) > 0$, it ought to be that

$$\begin{aligned} p_E(H_i) = p(H_i | E) = \frac{p(E|H_i)p(H_i)}{\sum ^n_{j=1} p(E|H_j)p(H_j)} \end{aligned}$$

So, if I entertain a set of hypotheses that form a partition, my posterior confidence in each hypothesis is obtained as follows: first, ask how likely my total evidence is given that hypothesis; second, weight the answer by how likely I thought the hypothesis was prior to receiving my most recent evidence; and, lastly, normalize the results.^2^

An updating rule is a function that takes a prior credence function and a body of total evidence and returns a recommended posterior credence function. If *p* is a prior, $\alpha$ is an updating rule, and *E* is some evidence, we write $p^\alpha _E$ for the posterior that $\alpha$ recommends to someone with prior *p* and total evidence *E*. We say that an updating rule $\beta$ is a Bayesian updating rule for *p* if, whenever $p(E) > 0$,

$$\begin{aligned} p^\beta _E(H_i) = \frac{p(E|H_i)p(H_i)}{\sum ^n_{j=1} p(E|H_j)p(H_j)} \end{aligned}$$

More briefly, we say that $\beta$ is Bayesian for *p* in this situation. In what follows, we’ll use ‘$\beta$’ whenever we are talking about a Bayesian updating rule.

We say that a prior *p* is regular if it gives positive credence to every possible world—that is, $p(w) > 0$, for all worlds *w*. If *p* is regular, there is just one updating rule that is Bayesian for it. However, if *p* is not regular, there will be many such rules, since Bayes’ Rule imposes no constraints on how you should update if you learn something to which you previously assigned zero credence.

Now, the credal version of inference to the best explanation tells us that $H_1$ is a better explanation for *E* than $H_2$ iff $p_E(H_1) > p_E(H_2)$, and Bayes’ Rule tells us that

$$\begin{aligned} p_E(H_1)> p_E(H_2) \text { iff } p(E|H_1)p(H_1) > p(E|H_2)p(H_2) \end{aligned}$$

So there are two straightforward ways to accommodate inference to the best explanation within Bayesianism:

1. Set $p(H_1) > p(H_2)$ and $p(E|H_1) \approx p(E|H_2)$. That is, assign a higher unconditional prior to more explanatory hypotheses.
2. Set $p(E|H_1) > p(E|H_2)$ and $p(H_1) \approx p(H_2)$. That is, assign a higher likelihood to the evidence conditional on the more explanatory hypothesis.

Either of these might account for my conclusion or my friend Robert’s when we saw that second shadow passing away from the bathroom that evening. It might have been that we roughly agreed on the likelihood of our evidence given each hypothesis, but disagreed on the prior probability of the hypothesis: Robert might just have been antecedently much more confident that ghosts exist, and much less confident that we were distracted enough to miss his mother’s shadow as she passed to go to the bathroom. Or we both might have agreed that it is very unlikely that ghosts exist and reasonably likely that we were distracted, but disagreed on how likely each hypothesis made our evidence: Robert might just have thought that, if ghosts were to exist, this is quite a likely way they’d show themselves. Or, of course, it might be a bit of both.

In general, we can better accommodate some cases of inference to the best explanation using (1), and some using (2), and some using a combination. You might, for instance, have two empirically equivalent hypotheses, such as the realist’s hypothesis that the external world exists and is as we perceive it to be ($H_1$) and the sceptic’s hypothesis that our experience of the external world is an illusion imposed on us by some powerful deceiver trying to trick us into thinking that it is the way we perceive it to be ($H_2$). In that case, providing neither is stronger than the other, it’s plausible that $p(E|H_1) = p(E|H_2)$. Indeed, regardless of their relative strength, if both hypotheses entail *E*, then $p(E|H_1) = 1 = p(E|H_2)$. In that case, we can only ensure that one receives higher posterior probability than the other by assigning it higher prior unconditional probability. So, if you want to use inference to the best explanation to justify your higher posterior in realism, you’d better set $p(H_1) > p(H_2)$. That is, you must use (1).

But sometimes (1) won’t do. I set an urn in front of you that contains three balls. I tell you that either two balls are violet and one green ($H_1$) or two balls are green and one violet ($H_2$). You will draw a ball at random, look at its colour, and update your credences in the two hypotheses in the light of your evidence. So there are two possible pieces of evidence you might receive: you might draw a violet ball ($E_1$) or you might draw a green one ($E_2$). Intuitively, $H_1$ explains $E_1$ better than $H_2$ does, while $H_2$ explains $E_2$ better than $H_1$ does. So, the credal version of inference to the best explanation demands that

$$\begin{aligned} p_{E_1}(H_1)> p_{E_1}(H_2) \text { and } p_{E_2}(H_2) > p_{E_2}(H_1) \end{aligned}$$

But we can’t ensure that only by setting $p(H_1) > p(H_2)$ or $p(H_2) > p(H_1)$. Instead, we must set $p(E_1 | H_1) > p(E_1 | H_2)$ and $p(E_2 | H_2) > p(E_2 | H_1)$. In fact, that seems reasonable anyway. Indeed, it is mandated by a norm that is often added to Probabilism and Bayes’ Rule to give a slightly stronger version of Bayesianism, namely, David Lewis’s Principal Principle (Lewis 1980).

**Principal Principle** It ought to be the case that

$$\begin{aligned} p(X\ |\ \text {the chance of}\ X\ \text {is}\ r) = r \end{aligned}$$

In the case we’re considering, the Principal Principle demands:

$$\begin{aligned} p(E_1 | H_1)= & {} \frac{2}{3} \qquad p(E_1 | H_2) = \frac{1}{3} \\ p(E_2 | H_1)= & {} \frac{1}{3} \qquad p(E_2 | H_2) = \frac{2}{3} \end{aligned}$$

If $p(H_1) = p(H_2)$, then by Bayes’ Rule we have:

$$\begin{aligned} p_{E_1}(H_1)> p_{E_1}(H_2) \text { and } p_{E_2}(H_2) > p_{E_2}(H_1) \end{aligned}$$

as we wished. And we obtained that using (2).

The upshot of the preceding discussion is that Bayesianism can accommodate much of what the credal version of inference to the best explanation demands.^3^ And, as Weisberg (2009) points out, it could go further and mandate it if we were to embrace a less subjectivist and more objectivist version of Bayesianism; one that limits the rational priors in such a way that, whenever $H_1$ better explains *E* than $H_2$ does, $p(H_1|E) > p(H_2|E)$.

Nonetheless, some think that this strategy does not go far enough. For instance, think again about the mystery urn from above. Suppose I have equal priors in the two hypotheses about the colour distribution in the urn; and suppose that $\beta$ is a Bayesian updating rule for my prior *p*.^4^ Then here are my posteriors if I draw a violet ball:

$$\begin{aligned} p_{E_1}(H_1) = p^\beta _{E_1}(H_1) = \frac{p(E_1 | H_1)p(H_1)}{p(E_1 | H_1) p(H_1) + p(E_1 | H_2)p(H_2)} = \frac{\frac{2}{3} \frac{1}{2}}{\frac{2}{3} \frac{1}{2} + \frac{1}{3} \frac{1}{2}} = \frac{2}{3} \end{aligned}$$

and

$$\begin{aligned} p_{E_1}(H_2) = p^\beta _{E_1}(H_2) = \frac{p(E_1 | H_2)p(H_2)}{p(E_1 | H_1) p(H_1) + p(E_1 | H_2)p(H_2)} = \frac{\frac{1}{3} \frac{1}{2}}{\frac{2}{3} \frac{1}{2} + \frac{1}{3} \frac{1}{2}} = \frac{1}{3} \end{aligned}$$

So $p_{E_1}(H_1) > p_{E_1}(H_2)$, as we hoped. But you might think that, while Bayes’ Rule results in higher posterior confidence in $H_1$ upon learning $E_1$, it doesn’t make that posterior confidence high enough. You might think that, upon seeing the violet ball, you should be even more confident in $H_1$ than Bayes’ Rule mandates, and even less confident in $H_2$. As I noted above, Bayes’ Rule says that my posterior confidence in each hypothesis from should be obtained by asking how likely the evidence is given that hypothesis, weighting that by how likely I thought the hypothesis was prior to receiving the evidence, and then normalizing the resulting credences. You might think instead that I should ask how likely the evidence is given the hypothesis, weight that by how likely I thought the hypothesis was prior to learning the evidence, *then add a little boost to that weighted likelihood if the hypothesis is one of best explanations of the evidence*, and then normalize.^5^ That is, instead of updating in line with Bayes’ Rule, we should update in line with what I’ll call the Explanationist’s Rule (with a specific boost *c*). I’ll define this more generally below, but for the moment, here is how it works in the particular case we’ve been considering. The Explanationist’s Rule (with boost *c*) says that your posteriors, upon learning $E_1$, should be

$$\begin{aligned} p_{E_1}(H_1)= & {} \frac{p(E_1 | H_1)p(H_1) + c}{p(E_1 | H_1)p(H_1) + p(E_1 | H_2)p(H_2) + c} \\= & {} \frac{\frac{2}{3} \frac{1}{2} + c}{\frac{2}{3} \frac{1}{2} + \frac{1}{3} \frac{1}{2} + c} = \frac{2 + 6c}{3 + 6c} \end{aligned}$$

That’s because $H_1$ best explains the evidence $E_1$—that is, $p(E_1 | H_1) > p(E_1 | H_2)$—and it therefore receives a boost in the numerator.

$$\begin{aligned} p_{E_1}(H_2)= & {} \frac{p(E_1 | H_2)p(H_2)}{p(E_1 | H_1)p(H_1) + p(E_1 | H_2)p(H_2) + c} \\= & {} \frac{\frac{1}{3} \frac{1}{2}}{\frac{2}{3} \frac{1}{2} +\frac{1}{3} \frac{1}{2} + c} = \frac{1}{3 + 6c} \end{aligned}$$

That’s because $H_2$ does not best explain $E_1$, and it therefore receives no boost.

So *c* is a boost that is awarded to the best explanation over and above what is already given by Bayes’ Rule. If $c = 0$, then the Explanationist’s Rule is just Bayes’ Rule. If $c >0$, then the explanationist demands that

- $p_{E_1}(H_1) = \frac{2 + 6c}{3 + 6c}$, which is greater than $\frac{2}{3}$, which is what Bayes’ Rule demands;
- $p_{E_1}(H_2) = \frac{1}{3 + 6c}$, which is less than $\frac{1}{3}$, which is what Bayes’ Rule demands.

The explanationist updating rule we just described is a particular case of the following rule, which Bas van Fraassen (1989, Chapter 6) sketched in his early discussion of the tension between inference to the best explanation and Bayesianism, and which Igor Douven (2013, 2021) has made precise and explored in great detail:

**Explanationist’s Rule (general)** If $H_1, \ldots , H_n$ is a set of mutually exclusive and exhaustive hypotheses, and $p(E) > 0$, then it ought to be that

$$\begin{aligned} p_E(H_i) = \frac{p(E |H_i) p(H_i) + f(H_i, E)}{\sum ^n_{j=1} \left( p(E|H_j)p(H_j) + f(H_j, E) \right) } \end{aligned}$$

where $f(H_i, E)$ rewards the hypothesis $H_i$ in some way that depends on the quality of the explanation it provides for the total evidence *E*.

In Douven’s version of the rule, each time you apply it, there is some fixed positive amount *c* of reward that we distribute evenly between the best explanations of the total evidence gathered so far. So, if there are *k* best explanations of *E*, then $f(H_i, E) = \frac{c}{k}$ if $H_i$ is among them, and $f(H_i, E) = 0$ if it is not.

**Explanationist’s Rule (Douven’s version)** Suppose $c \ge 0$. If $H_1, \ldots , H_n$ is a set of mutually exclusive and exhaustive hypotheses, and $p(E) > 0$, then it ought to be that

$$\begin{aligned} p_E(H_i) = \frac{p(E |H_i) p(H_i) + f_c(H_i, E)}{\sum ^n_{j=1} \left( p(E|H_j)p(H_j) + f_c(H_j, E) \right) } \end{aligned}$$

where $f_c(H_i, E) = \frac{c}{k}$ if $H_i$ is one of the *k* best explanations of *E* among $H_1, \ldots , H_n$, and $f_c(H_i, E) = 0$ if it is not.

Above, I explained that we say that an updating rule $\beta$ is Bayesian for *p* if, whenever $p(E) > 0$,

$$\begin{aligned} p^\beta _E(H_i) = \frac{p(E |H_i) p(H_i)}{\sum ^n_{j=1} \left( p(E|H_j)p(H_j) \right) } \end{aligned}$$

We also say that a rule $\varepsilon$ is explanationist for *p* with boost *c* if, whenever $p(E) > 0$,^6^

$$\begin{aligned} p^\varepsilon _E(H_i) = \frac{p(E |H_i) p(H_i) + f_c(H_i, E)}{\sum ^n_{j=1} \left( p(E|H_j)p(H_j) + f_c(H_j, E) \right) } \end{aligned}$$

As noted above, if $c = 0$, then Douven’s version of the Explanationist’s Rule coincides with Bayes’ Rule. But, typically, if $c > 0$, then it does not. So Bayes’ Rule conflicts with Douven’s version of explanationism. Which should we use? That is the question that will engage us for the rest of the paper. And it is a question of no small moment. Bayesianism is a central statistical tool in contemporary science, from epidemiology to particle detection; but inference to the best explanation is often advertised as a central component of the scientific method. If they do conflict and if we must choose one over the other, there will be work to do.

Van Fraassen defended Bayesianism against this version of explanationism by appealing to David Lewis’ betting argument for Bayes’ Rule. Douven has considered that argument, as well as other pragmatic considerations and also accuracy-based arguments for Bayes’ Rule. He thinks that none decisively establishes Bayes’ Rule, and presents considerations in favour of the non-Bayesian explanationist rule, at least in certain situations. His goal is to reject the dominance of Bayesianism, rather than to establish the dominance of explanationism. He allows that Bayes’ Rule may be the right way to go in certain situations, but sees no reason to think that’s always the case. In the remainder of the paper, I’ll consider Douven’s arguments and describe further arguments in favour of Bayes’ Rule, one pragmatic and two purely epistemic. I’ll argue that they provide compelling responses to Douven’s concerns. I conclude that the dominance of Bayes’ Rule should continue.

## Pragmatic arguments for Bayes’ Rule

I’ll start in this section with the argument for Bayes’ Rule to which van Fraassen appealed when he first argued against non-Bayesian versions of inference to the best explanation (van Fraassen 1989, Chapter 6). I’ll then consider Igor Douven’s responses to that argument, and that will lead me to introduce a further pragmatic argument for Bayes’ Rule.

### Lewis’ sure loss argument for Bayes’ Rule

Van Fraassen learned the sure loss argument for Bayes’ Rule from David Lewis, who had presented it in a seminar at Princeton in the 1970s, but didn’t publish it himself until 1999 (Lewis 1999). It’s a betting argument of the sort that Frank Ramsey and Bruno de Finetti provided for Probabilism (Ramsey 1926; de Finetti 1937). Like that argument, it starts with the following claim about what bets your credences should lead you to accept: if your credence in a proposition *X* is *p*, then for any stake *S*, whether positive or negative and regardless how large, you are rationally required to accept any bet that gains you more than $\pounds (1-p) S$ if *X* is true and loses you less than $\pounds p S$ if *X* is false. Lewis then proves a mathematical theorem: if your updating rule is not Bayesian for your prior, then (i) there is a series of bets each of which your prior rationally requires you to accept and (ii) whichever evidence you receive, there is a series of bets each of which the posterior credences demanded by your updating rule will rationally require you to accept such that (iii) when you add up the payouts at any possible world of the prior bets along with the posterior bets at that world, you see that they will lose you money.^7^ Lewis then contends that planning to update in a way that makes you vulnerable to such a sure loss is irrational.

Douven provides three responses to van Fraassen’s argument:

1. First, he suggests that we can have the best of both worlds by setting our priors in such a way that following Bayes’ Rule when we update gives us posteriors that agree with the explanationist’s updating rule but avoid the sure loss (Douven 1999). We’ll consider this in Sect. 1.2.
2. Second, he argues that, while it is certainly a consideration against an updating rule that it renders you vulnerable to a sure loss, we cannot conclude that it renders you irrational without considering whether there are considerations in its favour that compensate for this flaw; and he argues that there are such considerations (Douven 2013, 2021). This is the topic of Sects. 1.3 and 1.4.
3. Third, he (in one paper together with Sylvia Wenmackers) suggests that we cannot establish any credal norm by paying attention only to pragmatic considerations. We must instead show that there is an epistemic flaw in updating rules other than Bayes’ Rule (Douven 2013; Douven and Wenmackers 2017; Douven 2021). That will bring us to the accuracy arguments in Sect. 2, and their extension into questions of social epistemology in Sect. 3 and choices between different intellectual trajectories in Sect. 4.

### Avoiding the sure loss

You are about to learn something. You know that it will be a proposition in the partition $E_1, \ldots , E_m$. You consider each of the mutually exclusive and exhaustive hypotheses $H_1, \ldots , H_n$. Now consider a prior credence function *p* and an updating rule $\alpha$. Together, these determine, for each possible piece of evidence $E_j$, a posterior credence function $p^\alpha _{E_j}$ that the rule $\alpha$ says you should adopt if you learn $E_j$. Then Douven explains that we can always find an alternative prior *q* such that updating *q* on $E_j$ using an updating rule $\beta$ that is Bayesian for *q* will agree with updating *p* on $E_j$ using $\alpha$. That is, $p^\alpha _{E_j} = q^\beta _{E_j}$, for each possible piece of evidence $E_j$. So, if you take *q* to be your prior credence function and $\beta$ to be your updating rule, then (i) you’ll have exactly the posteriors that you would have had if you’d taken *p* to be your prior and $\alpha$ to be your updating rule, but (ii) your prior and updating rule won’t be vulnerable to a sure loss argument. In this way, Douven hopes, the explanationist escapes van Fraassen’s objection to their updating rule. By adopting *q* instead of *p*, you get all the supposed advantages of the posteriors recommended to *p* by the explanationist rule, but none of the disadvantages of violating Bayes’ Rule and making yourself vulnerable to a sure loss.

Here’s the trick: first, pick your alternative priors in the different possible pieces of evidence; that is, pick $0< q(E_1), \ldots , q(E_m) < 1$; then set your alternative priors in the conjunctions of hypotheses with evidence as follows:

$$\begin{aligned} q(H_iE_j) = p^\alpha _{E_j}(H_i)q(E_j) \end{aligned}$$

That then completely determines your alternative prior credence function *q*, and it’s easy to show that, defined in this way, *q* is a probability function.^8^ What’s more, if $\beta$ is a Bayesian updating rule for *q*, then, since *q* gives positive credence to all possible pieces of evidence,

$$\begin{aligned} q^\beta _{E_j}(H_i) = q(H_i |E_j) = \frac{q(H_iE_j)}{q(E_j)} =\frac{p^\alpha _{E_j}(H_i)q(E_j)}{q(E_j)} = p^\alpha _{E_j}(H_i) \end{aligned}$$

as required. So, in particular, if $\varepsilon$ is an explanationist updating rule for some prior *p*, we can pick an alternative prior *q* in such a way that, if $\beta$ is a Bayesian updating rule for *q*, then $q^\beta _{E_j} = p^\varepsilon _{E_j}$, for any possible piece of evidence $E_j$. Providing we then use *q* as our prior, we can update in line with Bayes’ Rule, and thereby hopefully sidestep the sure loss argument against the explanationist.

However, pushing down the lump in the carpet here just causes it to pop up unwanted elsewhere. In this case, using this trick leads to a prior that violates the Principal Principle, the extra norm of Bayesianism that we met above.

Return once more to our urn and the case in which we draw just a single ball. $H_1$ says that the chance of drawing a violet ball is two-thirds, while $H_2$ says the same for drawing a green ball. $E_1$ is your evidence if you draw a violet ball, and $E_2$ is your evidence if you draw a green ball. The Principal Principle demands that, if *q* is your prior, then

$$\begin{aligned} q(E_1 |H_1) = \frac{2}{3} = q(E_2|H_2) \end{aligned}$$

But according to the construction of the prior we’ve just described,

$$\begin{aligned} q(E_1|H_1)= & {} \frac{q(H_1E_1)}{q(H_1E_1) + q(H_1E_2)} \\= & {} \frac{p^\varepsilon _{E_1}(H_1)q(E_1)}{p^\varepsilon _{E_1}(H_1)q(E_1) + p^\varepsilon _{E_2}(H_1)q(E_2)} \\= & {} \frac{\frac{2 + 6c}{3+6c}q(E_1)}{\frac{2 + 6c}{3+6c}q(E_1) + \frac{1}{3+6c}q(E_2)} \\= & {} \frac{(2 + 6c)q(E_1)}{(2+6c)q(E_1) + q(E_2)} \end{aligned}$$

and

$$\begin{aligned} q(E_2|H_2)= & {} \frac{q(H_2E_2)}{q(H_2E_1) + q(H_2E_2)} \\= & {} \frac{p^\varepsilon _{E_2}(H_2)q(E_2)}{p^\varepsilon _{E_1}(H_2)q(E_1) + p^\varepsilon _{E_2}(H_2)q(E_2)} \\= & {} \frac{\frac{2 + 6c}{3+6c}q(E_2)}{\frac{1}{3+6c}q(E_1) + \frac{2+6c}{3+6c}q(E_2)} \\= & {} \frac{(2 + 6c)q(E_2)}{q(E_1) + (2+6c)q(E_2)} \end{aligned}$$

Now, if *q* satisfies the Principal Principle, then $q(E_1 |H_1) =\frac{2}{3} = q(E_2|H_2)$. And if that’s the case, then it is easy to see from the equations we’ve just set down that $q(E_1) =q(E_2)$. But then, by those same equations,

$$\begin{aligned} q(E_1 | H_1) = \frac{2 + 6c}{3 + 6c} \text { and } q(E_2 | H_2) = \frac{2 + 6c}{3 + 6c} \end{aligned}$$

But then $q(E_1 |H_1) = \frac{2}{3} = q(E_2|H_2)$ only if $c = 0$. So *q* satisfies the Principal Principle only if $c=0$. So, if $\varepsilon$ is explanationist for *p* with boost *c*, and $c > 0$, there is no *q* that satisfies the Principal Principle and Bayesian updating rule $\beta$ for *q* such that $q^\beta _{E_i}(H_j) = p^\varepsilon _{E_i}(H_j)$ for $i, j = 1, 2$.

So, if we want to give an extra boost to the best explanation of our total evidence over and above what Bayes’ Rule already gives it, and we wish to avoid Lewis’ sure loss argument against violations of Bayes’ Rule, we must pick a prior that violates the Principal Principle. And while the Converse Dutch Book Theorem ensures that there is no sure loss argument against violations of the Principal Principle that satisfy Probabilism, there is an expected loss argument against it (Pettigrew 2020, Section 2.8). It turns on the following fact: if you violate the Principal Principle, there is a set of bets that your credences will require you to enter into such that, whatever the objective chances are, those chances will expect you to lose money from those bets.

Douven himself recognises that the prior he constructs to match with a non-Bayesian updating rule might leave it vulnerable to some sort of betting argument. But he contends that such vulnerability is no threat to your rationality. After all, you could see your sure loss or expected loss coming, and simply refuse to enter into the final bet that locks you in to that loss (Douven 1999, pp. S429–S434).

One problem with this response is that, if it works against the expected loss argument for the Principal Principle, it also works against the sure loss argument for Probabilism, since the sure loss there is just as visible to the person who violates Probabilism as it is to the imagined bookie. However, the real problem with Douven’s argument is that this ‘look before you leap’ strategy works against neither argument. Suppose that you satisfy Probabilism but violate the Principal Principle, which is what Douven’s strategy requires of you. And suppose that, faced with a decision problem, rationality requires you to choose by maximizing expected utility. Then it turns out that you should accept each bet offered in the expected loss argument for the Principal Principle, since each maximises expected utility for you; and this is true even if you take into account the bets that you’ve already accepted (Pettigrew 2020, Section 3.4). So even at the final stage of the expected loss argument, where there is just one more bet to consider, and you know what you’ve already accepted and you can see that accepting this final bet locks you in to an expected loss from the point of view of the chances, accepting it still has greater expected utility from the point of view of your credence function than rejecting it. So even if you do look before you leap, and even if you do see what awaits you should you leap, your credences still rationally require you to leap. Indeed, it is this that renders them irrational.

Let me end this discussion with a briefer, less technical reply to Douven’s first objection to van Fraassen’s argument against explanationism. Contrary to what he claims, Douven’s approach does in fact leave you vulnerable to a sure loss. Suppose you have prior *p* and you wish to update by an explanationist rule $\varepsilon$. Then, Douven says, you should switch to prior *q*, and then update by a Bayesian rule $\beta$. But, while he’s right that updating *q* using $\beta$ ensures that you are not vulnerable to a sure loss, moving from *p* to *q* in the first place, without receiving any new evidence that prompts such a shift, does leave you vulnerable in this way. After all, a degenerate case of Lewis’ sure loss result says that changing credences without gaining any new evidence leaves you vulnerable to a sure loss, since doing so violates Bayes’ Rule. So it seems that Douven’s trick does not help us in any case, whether or not we are concerned about violating the Principal Principle.^9^

### The expected pragmatic utility argument for Bayes’ Rule

This brings us to Douven’s second objection to van Fraassen’s argument. The sure loss argument for Bayes’ Rule presents vulnerability to a sure loss as a flaw that renders an updating rule irrational. But it is a very peculiar sort of flaw. On the one hand, when it manifests, it will lose you money for sure, and there is no limit to the amount of money it will lose you, since the stake of the bets may be set as high as you like. But, on the other hand, the set of choices you must face in order that the flaw becomes manifest is very specific and quite unlikely to arise. So, if you think other decision problems are more likely, and if the credences your updating rule bequeaths to you serve you better when you face those than the credences that Bayes’ Rule demands, then you might well think that this outweighs the flaw of vulnerability to a sure loss.

I’m very sympathetic to the starting point of this argument. I agree that vulnerability to a sure loss does not, on its own, render credences irrational. But I think the prospects are bleak for finding some virtue of alternative updating rules that compensates for this flaw. The reason is that the sure loss argument is not the only argument for Bayes’ Rule that appeals to how well your credences serve you as a basis for decision-making. In this section, I’ll describe another.

The argument I have in mind is due to Brown (1976) and it is perhaps best seen as a generalization of I. J. Good’s Value of Information Theorem (Good 1967).^10^ The set up is this. I am about to learn some evidence. After I learn this new evidence, I’ll face a decision—that is, I’ll have to choose between a set of available acts. I’ll make this choice by maximising expected utility from the point of view of my credences at that time. How, then, should I plan to update my credences, knowing that I’ll use them to make this decision? Good showed that, if your only two options are to use a Bayesian updating rule or to simply stick with your prior when the evidence comes in, then your prior expects the Bayesian rule to produce posteriors that guide your choice after the evidence comes in better than sticking with your prior does. Brown generalizes this by showing that your prior expects Bayesian rules to produce posteriors that guide your actions better than *any* available updating rule.

Suppose:

- Your prior is *p*;
- The evidence you’re about to receive will be a proposition from a partition ${\mathcal {E}}$. If *w* is a possible world, $E_w$ is the unique proposition in ${\mathcal {E}}$ that is true at *w*.
- $\alpha$ is an updating rule that tells you to adopt $p^\alpha _E$ if you start with *p* and learn *E* from ${\mathcal {E}}$. We write $p^\alpha _w$ for $p^\alpha _{E_w}$. That is, $p^\alpha _w$ is the posterior you would end up with if you were to update the prior *p* on the evidence you would receive from ${\mathcal {E}}$ at world *w*.^11^
- If *a* is an act and *w* is a possible world, *a*(*w*) is the utility of *a* at *w*.
- If *q* is a credence function, $a^q$ is an act that maximizes expected utility by the lights of *q*, so that, for all acts *a*,
  $$\begin{aligned} \sum _{w \in W} q(w)a^q(w) \ge \sum _{w \in W} q(w)a(w) \end{aligned}$$

Then the expected utility of updating your prior *p* using rule $\alpha$ is:

$$\begin{aligned} \mathrm{Exp}_p(\text {Use rule}\ \alpha ) = \sum _{w \in W} p(w) a^{p^\alpha _w}(w) \end{aligned}$$

Now, let $\beta$ be a Bayesian updating rule for your prior *p*. And take a possible world $w^*$. Then, by the definition of $a^{p^\beta _{w^*}}$, for any updating rule $\alpha$,

$$\begin{aligned} \sum _{w \in W} p^\beta _{w^*}(w) a^{p^\beta _{w^*}}(w) \ge \sum _{w \in W} p^\beta _{w^*}(w) a^{p^\alpha _{w^*}}(w) \end{aligned}$$

So, since $p^\beta _{w^*}(w) = \frac{p(w)}{p(E_{w^*})}$ if *w* is in $E_{w^*}$ and $p^\beta _{w^*}(w) = 0$ if *w* is not in $E_{w^*}$,

$$\begin{aligned} \sum _{w \in E_{w^*}} p(w) a^{p^\beta _{w^*}}(w) \ge \sum _{w \in E_{w^*}} p(w) a^{p^\alpha _{w^*}}(w) \end{aligned}$$

But of course, if *w* is in $E_{w^*}$, then $E_{w} = E_{w^*}$ and $p^\beta _{w} = p^\beta _{w^*}$ and $p^\alpha _{w} = p^\alpha _{w^*}$. So

$$\begin{aligned} \sum _{w \in W} p(w) a^{p^\beta _{w}}(w) \ge \sum _{w \in W} p(w) a^{p^\alpha _{w}}(w) \end{aligned}$$

So

#### Theorem 1

(Expected pragmatic argument) For any prior *p*, any updating rule $\beta$ that is Bayesian for *p*, and any updating rule $\alpha$,

$$\begin{aligned} \mathrm{Exp}_p(\text {Use rule}\ \beta )\ge \mathrm{Exp}_p(\text {Use rule}\ \alpha ) \end{aligned}$$

And, if there is a world *w* such that *(i)* $a^{p^\beta _w} \ne a^{p^\alpha _w}$ and *(ii)* $p(w) > 0$, then this inequality is strict.

That is, if you give any prior credence to ending up with a posterior that chooses differently from how a posterior obtained from a Bayesian rule $\beta$ will choose, then your prior expects updating using $\beta$ to be strictly better. So, if we must make a choice after receiving some evidence, our prior expects us to make that choice best if we choose using the posteriors we get by updating in line with Bayes’ Rule.

Of course, we are not often in the precise situation covered by this result. Rarely do we know which decisions we will face using the posteriors that our updating rule bestows on us when we deploy it on our next piece of evidence. What’s more, an updating rule doesn’t just give you the credences you will use to make decisions after you receive this piece of evidence. It also gives you the credences you will update when you receive the next piece of evidence after that. And then the credences you will update when you receive the next piece of evidence after that. And so on. So we should be concerned not only with the choices that our updated credences mandate, but also the choices that our updated updated credences mandate and our updated updated updated credences, and so on.

Fortunately, Brown’s reasoning goes through even for this more complex but more realistic situation, provided we grant a certain assumption, which we’ll explain below. Here’s the setup. Suppose *p* is your prior. Suppose $t_1, \ldots , t_n$ are the times during your epistemic life. For each $1 \le i \le n$,

- Your total evidence at $t_i$ is a proposition in the partition ${\mathcal {E}}_i$. Let $E_{w, i}$ be the total evidence from ${\mathcal {E}}_i$ that you will have at time $t_i$ at world *w*.^12^
- If $\alpha$ is an updating rule and *w* is a possible world, $p^\alpha _{w,i}$ is the credence function you reach in world *w* by time $t_i$ if you start with prior *p* and successively apply $\alpha$ to the total evidence you’ll have at that world at each time $t_1, \ldots , t_i$.
- The decision problem you will face at $t_i$ comes from the set ${\mathcal {D}}_i$. We can assume without loss of generality that you just face a single decision problem at each time $t_i$. If you face two, we just combine them into a single composite one.^13^ Let $D_{w, i}$ be the decision problem in ${\mathcal {D}}_i$ that you face at time $t_i$ in world *w*.
- $0< \lambda _i < 1$ is the weight that records how much you care about the pragmatic utility your credences obtain for you at time $t_i$.
- Given credence function *q* and decision problem *D*, let $a^q_D$ be an act in *D* that maximises expected utility from the point of view of *q*.

Then:

$$\begin{aligned} \mathrm{Exp}^\Lambda _p(\text {Use rule}\ \alpha ) = \sum _{w \in W} p(w) \sum _{t_i} \lambda _i a^{p^\alpha _{w, i}}_{D_{w, i}}(w) \end{aligned}$$

Now we introduce the assumption we must make if we are to extend Brown’s proof: for any time, the evidence you receive at that time tells you what decision problem you will face at that time. That is, if you receive the same evidence at two different worlds at a given time, you face the same decision problem at those worlds. In symbols: for any time $t_i$ and for all worlds $w, w'$, if $E_{w, i} = E_{w', i}$, then $D_{w, i} = D_{w', i}$. Assuming that, we can prove:

$$\begin{aligned} \mathrm{Exp}^\Lambda _p(\text {Use rule}\ \beta ) \ge \mathrm{Exp}^\Lambda _p(\text {Use rule}\ \alpha ) \end{aligned}$$

After all, for any world $w^*$ and any time $t_i$, by the definition of $a^{p^\beta _{w^*, i}}_{D_{w^*, i}}$,

$$\begin{aligned} \sum _{w \in W} p^\beta _{w^*, i}(w) a^{p^\beta _{w^*, i}}_{D_{w^*, i}}(w) \ge \sum _{w \in W} p^\beta _{w^*, i}(w) a^{p^\alpha _{w^*, i}}_{D_{w^*, i}}(w) \end{aligned}$$

So

$$\begin{aligned} \sum _{w \in W} p(w | E_{w^*, i}) a^{p^\beta _{w^*, i}}_{D_{w^*, i}}(w) \ge \sum _{w \in W} p(w | E_{w^*, i}) a^{p^\alpha _{w^*, i}}_{D_{w^*, i}}(w) \end{aligned}$$

So,

$$\begin{aligned} \sum _{w \in E_{w^*, i}} p(w) a^{p^\beta _{w^*, i}}_{D_{w^*, i}}(w) \ge \sum _{w \in E_{w^*, i}} p(w) a^{p^\alpha _{w^*, i}}_{D_{w^*, i}}(w) \end{aligned}$$

But of course, if *w* is in $E_{w^*, i}$, then $E_{w, i} = E_{w^*, i}$. So, $p^\beta _{w, i} = p^\beta _{w^*, i}$ and $p^\alpha _{w, i} =p^\alpha _{w^*, i}$. What’s more, by our assumption, $D_{w, i} =D_{w^*, i}$. So

$$\begin{aligned} \sum _{w \in W} p(w) \sum _{t_i} \lambda _i a^{p^\beta _{w, i}}_{D_{w, i}}(w) \ge \sum _{w \in W} p(w) \sum _{t_i} \lambda _i a^{p^\alpha _{w, i}}_{D_{w, i}}(w) \end{aligned}$$

And thus

#### Theorem 2

(Longitudinal expected pragmatic argument) For any prior *p*, any updating rule $\beta$ that is Bayesian for *p*, and any updating rule $\alpha$,

$$\begin{aligned} \mathrm{Exp}^\Lambda _p(\text {Use rule}\ \beta ) \ge \mathrm{Exp}^\Lambda _p(\text {Use rule}\ \alpha ) \end{aligned}$$

And, if there is a time $t_i$ and a world *w* such that *(i)* $a^{p^\beta _{w, i}} \ne a^{p^\alpha _{w, i}}$ and *(ii)* $p(w) > 0$, then this inequality is strict.

That is, if you give any prior credence to ending up at some point with a posterior that chooses differently from how the Bayesian’s posterior will choose at that point, then your prior expects updating using $\beta$ to be strictly better.

The problem with the sure loss argument for Bayes’ Rule is that it declares any alternative updating rule irrational just because there is a very specific decision problem you might face where your priors, together with the credences issued by that updating rule, serve you very badly indeed—to wit, they lead you to accept a sure loss. Douven’s worry is that, while this is certainly a strike against non-Bayesian updating rules, it is a shortcoming for which they might compensate in other ways. The foregoing expected pragmatic utility argument pours cold water on that hope. Whichever series of decision problems you might face at whatever stage of your epistemic life, and almost whatever prior credences you have in facing those decisions, you will be served best by updating using Bayes’ Rule. Or at least that is what your prior expects.

Now Douven notes that we surely care more about the *actual* pragmatic utility of adopting a particular updating rule than about its *expected* pragmatic utility. So does the foregoing argument tell us nothing until we find out which rule maximizes actual pragmatic utility? Surely not. This objection mistakes the reason we care about expected pragmatic utility. We care about it precisely because we care about actual pragmatic utility. It is our best way of choosing options when maximizing actual pragmatic utility is our aim but our ignorance of what the actual world is like prevents us from maximizing that directly. When I have a headache and choose which painkiller to take, I ask myself which will minimize my expected pain. I do this not because I care about expected pain in itself, but because I care about my actual pain, and I think minimizing expected pain is my best shot at minimizing that.

If we know more about the actual world than is encoded in our prior, then we should incorporate that new information into our prior and then do whatever maximizes expected pragmatic utility from the point of view of this new updated prior. And, again, the advice will be to follow Bayes’ Rule, but this time applied to our updated prior. It is no surprise that, if we know more about the actual world than our prior does, we can find updating rules that actually outperform what our prior expects to do best. If I know that, in fact, the urn contains two violet balls and one green ball, while my prior assigns only credence 0.5 to that hypothesis, then I can simply update by setting my credence in that hypothesis to 1, regardless of the further evidence I observe, and doing so will actually outperform Bayesian updating as applied to my prior. But that is no objection to the expected pragmatic utility argument for Bayes’ Rule.

### Getting to the truth faster

Nonetheless, Douven thinks there is a pragmatic virtue of the explanationist’s rule that might save it from irrationality. He does not consider the expected pragmatic utility argument just described, so we can’t know whether he thinks those virtues outweigh the flaws that argument identifies, but let’s consider the matter ourselves.

In short, Douven claims that an explanationist updating rule might lead us to converge to the truth more quickly than a Bayesian updating rule (Douven 2013, 2021). He uses computer simulations of their performance to support this conclusion. The example he uses is a slight variation of the urn case we described above. Instead of three balls, there are ten in this urn; but, as before, all are coloured violet or green; and, as before, you know nothing of the distribution. There might be no violet and ten green ($H_0$), one violet and nine green ($H_1$), and so on up to nine violet and one green ($H_9$), and ten violet and no green ($H_{10}$). So $H_i$ is the hypothesis that there are exactly *i* violet balls in the urn.

Throughout, Douven assumes that your prior is regular, and so there is just one Bayesian rule for it and just one explanationist rule with a given boost.

Douven begins by making precise what he means by converging to the truth. He sets a threshold—in particular, he picks 0.9, though it seems plausible that we’d see the same phenomenon for other values. And he says that a credence function leads us to assert the truth of the hypothesis if it assigns it a credence that lies above that threshold. Then, for each possible composition of the urn, he asks a computer to simulate drawing a ball, looking at it, and replacing it 500 times in a row; and he asks it to do that 1000 times. He also asks the computer to start with two uniform priors over the eleven hypotheses $H_1, \ldots , H_{10}$ about the urn’s contents and then to update one of them after each draw using Bayes’ Rule and to update the other after each draw using the explanationist’s rule. Then, for each draw from the urn, he looks at the proportion of those 1000 sequences of 500 draws at which updating using the Bayesian rule leads to credences in the true hypothesis that first cross the 0.9 threshold at that draw, and the proportion at which updating using the explanationist rule leads to credences in the true hypothesis that first cross the 0.9 threshold at that draw. That is, for each draw, he asks how likely it is that the Bayesian rule ‘gets it right’ for the first time at that draw, and how likely it is that the explanationist rule ‘gets it right’ for the first time at that draw. And he asks for which of the two updating rules does the draw at which it is most likely to first ‘get it right’ occur earliest. For each possible hypothesis about the composition of the urn, his simulations show that it is the explanationist rule. He then asks the same question but not for the draw with the highest chance of your rule getting it right, but for the draw with the highest chance of your rule getting it right *and remaining right*, where by that he means that the credence in the true hypothesis crosses the threshold *and stays there for the remainder of the draws*. And again it is the explanationist rule.

One problem with this argument is that it isn’t clear how impressive explanationism’s victory is here. Consider, for instance, the following update rule. Whatever prior it is given, it recommends no change until you have seen ten draws from the urn. Then, if the first ten draws contain exactly *i* purple balls, assign credence 1 to hypothesis $H_i$, which says the urn contains exactly *i* purple balls, and 0 to all the others. Then never change your credences again, whatever further draws you witness. Now, for each hypothesis $H_i$, run the same 1000 versions of the sequence of 500 draws from the urn. At which draw is it most likely this rule will lead to credence greater than 0.9 in the true hypothesis for the first time? And at which draw is it most likely to do that and then never fall below that threshold again? Well, it’s the tenth draw for both, of course. Granted, it might not get it right at that toss. And indeed if it doesn’t it never will. But if it’s going to do it, it’s going to do it then. Indeed, consider the rule that behaves exactly like this, but instead of assigning credence 1 to $H_i$ when you see *i* purple balls among the first ten draws, it instead assigns 1 to $H_1$ if no purple balls are drawn, it assigns 1 to $H_2$ if exactly one purple ball is drawn, it assigns 1 to $H_3$ if exactly two purple balls are drawn, and so on until it assign 1 to $H_{10}$ if exactly nine purple balls are drawn, and 1 to $H_0$ if ten purple balls are drawn. Again, it’s most likely to get it right for the first time at the tenth draw, and most likely to get it right and stay right at that same draw. So these two rules outperform both the Bayesian rule and the explanationist rule according to the measure that Douven introduces. But these are, of course, terrible rules. And that suggests that we should not care much about this measure of convergence to the truth.

Now, you might wonder what all of this has to do with pragmatic utility. Here is Douven:

[I]magine that the hypotheses concern some scientifically interesting quantity—such as the success rate of a medical treatment, or the probability of depressive relapse—rather than the bias of a coin, and the tosses are observations or experiments aimed at determining that quantity. Which researcher would not want to use an update rule that increases her chances of being in a position to make public a scientific theory, or a new medical treatment, before the (Bayesian) competition is? (Douven 2021, p. 103)^14^

Well, here is one answer: a researcher who wishes to update in a way that gives her posterior credences that her prior expects will lead her to the best choice when she uses them to face decisions. And Brown’s expected pragmatic utility argument from above says that Bayesian rules do this. We might suppose that the researcher will receive a stream of data, some parcel at each of a number of successive times. At each time, they’ll face the same decision: make the new treatment public, or don’t. The decision whether to announce a new treatment is always difficult. If you announce early and it’s safe and effective, you prevent lots of suffering. If you announce early and it’s safe but ineffective, you prevent no suffering, but equally you cause none, but perhaps you precipitate some loss of faith in medical science. And so on. So our researcher tries to quantify the utility of these different outcomes, assign credences to the different possibilities, and choose. But if they know that, at each time, they’ll choose whether or not to make their treatment public by maximising their expected utility by the lights of their credences at that time, we know from the result of the previous section that they should update using a Bayesian rule. They will expect their future choices to have lower utility if they update in any other way.

This point is relevant also to a game Douven describes that pits the Bayesian against the explanationist, and is intended as another way to find out which converges to the truth faster. Again, in this game, balls are drawn and replaced from an urn containing ten balls, each violet or green; again, we don’t know how many of each colour. After each draw, the Bayesian and the explanationist update the uniform prior using their favoured rule. And, at each point, they raise their hand if their credence in one of the hypotheses has risen above 0.9. The scoring system is then somewhat elaborate. Before we consider it, let’s consider a slightly different, but much simpler system. If a player does not raise their hand on a given draw, they add 0 points to their total; if they do raise it and the hypothesis in which their credence is above 0.9 is true, they receive 1 point; if they raise it and the hypothesis is false, they lose 1 point. Now, I don’t know which player will typically win this game, but it wouldn’t surprise me at all if it is the explanationist. Nonetheless, I don’t think this would tell against the Bayesian. After all, given that reward structure, raising your hand exactly when your credence in a hypothesis rises above 0.9 is just not what the Bayesian would choose to do. Rather, they would raise their hand whenever they would maximise expected utility by doing so, and that would be whenever their credence in a hypothesis rose above 0.5. After all, they would then have greater than 0.5 credence that they would obtain 1 point by raising their hand, and less than 0.5 credence that they would lose 1 point by doing so. And so raising their hand will have positive expected utility, which is greater than the guaranteed utility of 0 that keeping their hand lowered will have.

So it is no great criticism of Bayesianism that their credences lead them to have fewer points at the end of a game in which they wouldn’t have chosen to play the way they were forced to play. One lesson from the literature on epistemic consequentialism is that, for any epistemic behaviour whatsoever, however rational, we can create a way of scoring epistemic states on which it performs poorly (Jenkins 2007; Greaves 2013; Ahlstrom-Vij and Dunn 2018; Elstein and Jenkins 2020). Imagine this game: at successive stages, I work through the propositions to which you assign credences and I ask you to report your credence in that proposition and in its negation. The points you receive at each turn is the difference between 1 and the sum of your credences in that turn’s proposition and its negation. Many incoherent agents will win this game against a coherent agent. But that does not tell against Probabilism.

Now, in Douven’s version of this game, things are slightly more complicated. Nonetheless, the same problem arises. For him, the points you receive at a given draw depend not only on your credences, but also on your opponent’s. Here are the possibilities:

**Table:** 

	Raise & Right	Raise & Wrong	Don’t Raise
Raise & Right	1,1	0,2	0,1
Raise & Wrong	2,0	0,0	1,0
Don’t Raise	1,0	0,1	0,0

In the simpler version of the game, when each player considered whether raising their hand or keeping it lowered would maximise expected utility, they had only to consider their credences in the different hypotheses, since their score was dependent only on which of those was true. In this game, since the points they receive depend not only on which hypothesis is true but also on whether their opponent raises their hand as well, they must attend to their opponent’s credences too. But the problem with the argument is the same: in each case, neither Bayesian nor explanationist would choose to raise their hand exactly when their credence in a hypothesis rises above 0.9. So it is no pragmatic argument against the Bayesian that their update rule, coupled with a non-Bayesian decision rule governing their play in this game, performs worse than the explanationist updating rule coupled with the same non-Bayesian decision rule.

## Accuracy arguments for Bayes’ Rule

So much, then, for the practical benefits of updating using either a Bayesian rule or an explanationist alternative or some other sort of rule entirely. Alongside these practical arguments for Bayes’ Rule—Lewis’ sure loss argument and Brown’s expected utility argument—there are also purely epistemic arguments. These appeal not to how well the updated credences guide your actions, but how accurately they represent the world. The idea is this: just as full beliefs represent the world accurately by being true, a credence in a true proposition is more accurate the higher it is and a credence in a false proposition is more accurate the lower it is. Assuming veritism, which says that accuracy is the fundamental source of purely epistemic value, we can give accuracy arguments for norms that govern credences by showing that, if you violate the norm, your credences are somehow suboptimal from the point of view of accuracy. Accuracy arguments have been given for Probabilism (Joyce 1998, 2009; Pettigrew 2016a), Bayes’ Rule (Greaves and Wallace 2006; Leitgeb and Pettigrew 2010; Briggs and Pettigrew 2020), the Principal Principle (Pettigrew 2013), the Principle of Indifference (Leitgeb and Pettigrew 2010; Pettigrew 2016b), and norms governing peer disagreement (Levinstein 2015), higher-order evidence (Schoenfield 2016), and the permissibility or impermissibility of rationality (Horowitz 2014; Schoenfield 2019), among many others. If the accuracy arguments for Bayes’ Rule succeed, they tell against explanationism. So in this section we consider them and ask whether they do, indeed, succeed.

The first accuracy argument for Bayes’ Rule closely mirrors the expected pragmatic argument, and indeed it appeals to almost exactly the same mathematics; the second accuracy argument is a little different. Just as the expected pragmatic argument showed that you will expect the credences demanded by a Bayesian rule to serve you best as a guide to future decision-making, so the expected epistemic utility argument shows that you will expect the credences demanded by such a rule to most accurately represent the world. That’s the first accuracy argument for Bayes’ Rule. The second, which we call the accuracy dominance argument, shows this: if you plan to update your prior by anything other than a Bayesian rule, then there will be an alternative prior and an alternative updating rule that, taken together, more accurately represent the world than your prior and your updating rule do—that is, taken together, your prior and updating rule will be accuracy dominated.

At the heart of accuracy arguments for credal norms lie the measures of accuracy. Each such measure ${\mathfrak {A}}$ takes a credence function *q* and a possible world *w* and returns ${\mathfrak {A}}(q, w)$, which measures the accuracy of *q* at *w*. We assume that every legitimate measure of accuracy boasts the following two properties:

**Continuity of** $${\mathfrak {A}}$$

For each world *w*, ${\mathfrak {A}}(q, w)$ is a continuous function of *q*.

**Strict Propriety of** ${\mathfrak {A}}$

${\mathfrak {A}}$ is strictly proper. That is, for any two probability functions $p \ne q$, *p* expects itself to be more accurate than it expects *q* to be. That is,

$$\begin{aligned} \mathrm{Exp}_p({\mathfrak {A}}(p)) > \mathrm{Exp}_p({\mathfrak {A}}(q)) \end{aligned}$$

That is,

$$\begin{aligned} \sum _{w \in W} p(w) {\mathfrak {A}}(p, w) > \sum _{w \in W} p(w) {\mathfrak {A}}(q, w) \end{aligned}$$

Both properties are assumed in nearly all discussions of epistemic utility, and I won’t rehearse arguments in their favour here.^15^ There are many many accuracy measures that boast them both, but it will suffice to mention just the most popular pair. Suppose *p* is a credence function defined on a set of propositions ${\mathcal {F}}$. Given a proposition *X* in ${\mathcal {F}}$ and a possible world *w*, let $w(X) = 1$ if *X* is true at *w* and $w(X) = 0$ if *X* is false at *w*. Then:

**Brier score** First, define the quadratic scoring rule:

- $${\mathfrak {q}}(0, x) = -x^2$$
- $${\mathfrak {q}}(1, x) = -(1-x)^2$$

Then define the Brier score of *p* at *w*:

$$\begin{aligned} {\mathfrak {B}}(p, w) = \sum _{X \in {\mathcal {F}}} {\mathfrak {q}}(w(X), p(X)) \end{aligned}$$

**Additive log score** First, define the logarithmic scoring rule:

- $${\mathfrak {l}}(0, x) = -x$$
- $${\mathfrak {l}}(1, x) = \log x - x$$

Then define the additive log score of *p* at *w*:

$$\begin{aligned} {\mathfrak {L}}(p, w) = \sum _{X \in {\mathcal {F}}} {\mathfrak {l}}(w(X), p(X)) \end{aligned}$$

We then have the following mathematical results. The setup is the same as in the pragmatic case, except that we don’t assume that there are any decision problems you might face using the credences you obtain from your prior and your updating rule. So $t_1, \ldots , t_n$ are the times in your epistemic future, and $t_0$ is the time at which you have your prior. At each time $t_i$ your total evidence will come from the partition ${\mathcal {E}}_i$. ${\mathfrak {A}}_i$ measures the accuracy of your credences at time $t_i$. And $0< \lambda _i < 1$ is the weight that encodes how much you care about the accuracy of your credences at $t_i$. Then we define the longitudinal accuracy of an updating rule $\alpha$ applied to a prior *p* as follows:

$$\begin{aligned} {\mathfrak {A}}(\alpha , w) = \lambda _1 {\mathfrak {A}}_1(p^\alpha _{w,1}, w) + \cdots + \lambda _n {\mathfrak {A}}_n(p^\alpha _{w, n}, w) \end{aligned}$$

Then,

### Theorem 3

(Longitudinal expected accuracy argument) For any prior *p*, any updating rule $\beta$ that is Bayesian for *p*, and any updating rule $\alpha$,

$$\begin{aligned} \sum _{w \in W} p(w) {\mathfrak {A}}(\beta , w) \ge \sum _{w \in W} p(w) {\mathfrak {A}}(\alpha , w) \end{aligned}$$

And the inequality is strict if there is a world *w* and a time $t_i$ such that *(i)* $p^\beta _{w, i} \ne p^\alpha _{w, i}$ and *(ii)* $p(w) > 0$.

This is the accuracy analogue of the expected pragmatic utility argument for Bayes’ Rule. It generalizes the argument by Hilary Greaves and David Wallace that applies when you learn just once (Oddie 1997; Greaves and Wallace 2006). It shows that any prior will expect a rule that is Bayesian for that prior to produce more accurate credences than it expects any other sort of rule to produce.

And we define the longitudinal accuracy of a prior and updating rule together as follows:

$$\begin{aligned} {\mathfrak {A}}((p, \alpha ), w) = \lambda _0 {\mathfrak {A}}_0(p, w) + \lambda _1 {\mathfrak {A}}_1(p^\alpha _{w,1}, w) + \ldots + \lambda _n {\mathfrak {A}}_n(p^\alpha _{w, n}, w) \end{aligned}$$

But then it is possible to establish the following result, which generalises an argument due to R. A. Briggs and me, which has recently been corrected and improved by Michael Nielsen (Briggs and Pettigrew 2020; Nielsen 2021).

### Theorem 4

(Longitudinal accuracy dominance argument) Suppose each ${\mathfrak {A}}_i$ is continuous and strictly proper.

(I) If *p* is a prior and $\alpha$ is an updating rule that is not Bayesian for *p*, then there is a prior *q* and an updating rule $\beta$ that is Bayesian for *q* such that, for all *w*,
  $$\begin{aligned} {\mathfrak {A}}((q, \beta ), w) > {\mathfrak {A}}((p, \alpha ), w) \end{aligned}$$
(II) If *p* is your prior and $\beta$ is an updating rule that is Bayesian for *p*, then there is no prior *q* and updating rule $\alpha$ such that, for all *w*,
  $$\begin{aligned} {\mathfrak {A}}((g, \alpha ), w) > {\mathfrak {A}}((p, \beta ), w) \end{aligned}$$

That is, if you are picking priors and updating rules together, you will avoid accuracy dominance only if you pick a prior and a Bayesian rule for it. That is, if you pick a prior together with any other sort of rule, there is an alternative prior such that picking that together with a Bayesian rule would produce more total accuracy for you in all worlds.

The difference between the two accuracy results is the scope of the norm they seek to establish. The expected accuracy result tries to establish a narrow scope norm: if you *p* is your prior, then you ought not to pick an updating rule $\alpha$ that is not Bayesian for *p*. Its form: If *A*, then it ought to be that *B*. The accuracy dominance result wishes to establish a wide scope norm: you ought not to have a prior *p* and an updating rule $\alpha$ that is not Bayesian for *p*. Its form: It ought to be that, if *A*, then *B*.

Before we move on to Douven’s simulation results concerning the accuracy of the two competing updating rules, it’s worth noting how these results answer one of his concerns. He writes:

The general problem for the inaccuracy-minimization approach this points to is that [minimizing accuracy] permits of a number of different interpretations. For instance, it can be interpreted as demanding that every single update minimize expected inaccuracy [...] or that every update minimize actual inaccuracy, or that every update be aimed at realizing the long-term project of coming to have a minimally inaccurate representation of the world, even if individual updates do not always minimize inaccuracy or expected inaccuracy. (Douven 2021, p. 108)

In the longitudinal versions of the expected accuracy and accuracy dominance arguments we just described, we needn’t weight all moments in the individual’s epistemic life equally. If we are interested primarily in our long-run accuracy, we can give the lion’s share of the weight to later points in our life. On the other hand, if we want to maximise getting quick results, perhaps in time for a big decision at the end of the week, we can shift it all to the times that lie within the next few days. But whatever we do, the results will be the same: Bayesian rules are the best.^16^

Nonetheless, Douven thinks there is still a sense in which explanationism does better than Bayesianism from an accuracy point of view (Douven 2021, Section 4.3). Again, he uses computer simulations to make his point. This time, he considers sequences of 1000 draws from our urn. For each hypothesis, he asks his computer to produce 1000 such sequences and to update one uniform prior using the Bayesian rule for it after each draw, and one uniform prior using the explanationist rule.^17^ After 100, 250, 500, 750, and 1000 draws, he compares the accuracy of the results of these two updating rules using the Brier score. He shows that, for any hypothesis and any one of these five staging posts, the explanationist is more likely to have produced the more accurate credences of the two; and much more likely for the more extreme hypotheses.

How can we reconcile this fact with the expected accuracy argument above? Well, as Douven himself notes, in those many cases where the explanationist does better than the Bayesian, they do only slightly better, while in the cases where the Bayesian prevails, they do a lot better. So, in expectation, the Bayesian rule is superior, even though in most cases, the explanationist rule is better. Douven contends that, while this doesn’t tell decisively in favour of the of the explanationist, it does undermine the claim that accuracy considerations tell decisively in favour of Bayes’ Rule. If we care about being more accurate most of the time, rather than having greatest expected accuracy, we should be explanationists. And caring in this way is reasonable.

This is an interesting result, and it should give Bayesian’s pause. But is it really reasonable to care about the probability of comparative performance and ignore the distribution of absolute performance? Let’s think how that pattern of caring would play out in a practical decision. Suppose, for instance, I think there are three possible outcomes of a new treatment for a particular medical condition: on the first, it alleviates the condition a very small amount; on the second, it alleviates the condition a small amount; on the third, it exacerbates the condition greatly and indeed produces new complications far far worse than the original condition. If I’m equally confident in these three possibilities, it doesn’t seem at all reasonable to favour administering the drug, even though doing so is better in the majority of cases. Indeed, the very purpose of expected utility theory is to give us the means to navigate this sort of problem.

You might reasonably respond to this by pointing out that many decision theorists now think that maximizing expected utility isn’t rationally mandated. Responding to examples like the Allais paradox, they hold that it is rationally permissible to use decision rules that give greater weight to worst-case scenarios than expected utility gives, and it is rationally permissible to use decision rules that give greater weight to best-case scenarios than expected utility gives (Allais 1953; Quiggin 1993). Perhaps the best example of such rules are given by Lara Buchak’s risk-weighted expected utility theory (Buchak 2013). I don’t have a settled view on the rational permissibility of these rules. But I do know that they all obey the dominance or Pareto principle, which says that an option that is worse in every possible state of the world should be dispreferred. And of course the accuracy dominance result appeals not to expected utility theory, but only to such a dominance principle. Recall: the accuracy dominance argument for Bayes’ Rule notes that, if you plan to update your prior by any other sort of rule, there is an alternative prior and an alternative updating rule that is guaranteed to have greater total accuracy than your prior and your updating rule. Since the risk-sensitive decision rules to which I just referred will always prefer one option to another if the first is better at all worlds, they will prefer the pairing of the alternative prior and updating rule to the pairing of your prior and updating rule. So they will consider your choice of updating rule irrational.

## Updating in a social setting

So far, the setting for the stand offs between Bayes’ Rule and explanationism has been the epistemology of individuals. That is, we have considered only the single solitary agent collecting evidence directly from the world and updating on it. But of course we often receive evidence not directly from the world, but indirectly through the opinions of others. I learn how many positive SARS-CoV-2 tests there have been in my area in the past week not my inspecting the test results myself but by listening to the local health authority. In their 2017 paper, ’Inference to the Best Explanation versus Bayes’s Rule in a Social Setting’, Douven joined with Sylvia Wenmackers to ask how Bayes’ Rule and explanationism fare in a context in which some of my evidence comes from the world and some from learning the opinions of others, where those others are also receiving some of their evidence from the world and some from others, and where one of those others from whom they’re learning might be me (Douven and Wenmackers 2017). As for Douven’s studies in the individual setting, Douven and Wenmackers conclude in favour of explanationism. Indeed, their conclusion in this case is considerably stronger than in the individual case:

The upshot will be that if agents not only update their degrees of belief on the basis of evidence, but also take into account the degrees of belief of their epistemic neighbours, then the noted advantage of Bayesian updating [from (Douven 2013)] evaporates and explanationism does better than Bayes’s rule on every reasonable understanding of inaccuracy minimization. (Douven and Wenmackers 2017, pp. 536–7)

As before, I want to stick up for Bayes’ Rule. As in the individual setting, I think this is the update rule we should use in the social setting.

In the individualistic cases we considered above, there’s a single urn containing a particular number of violet and green balls. The individual draws and replaces balls one at a time, and updates their credences about the balls in the urn on the basis of those observations. In the social setting case, we assume each individual has an urn, and each of these urns has the same distribution of violet and green balls in it. So, again, the hypotheses in question are $H_0, \ldots , H_{10}$, where $H_i$ says that each individual’s urn contains exactly *i* violet balls. As before, we assume each individual has the same uniform prior over the hypotheses, and obeys the Principal Principle. Douven and Wenmackers then assume that things proceed as follows:

- Step (i)  Each member draws and replaces a ball from their urn a certain number of times. This produces their worldly evidence for this round.
- Step (ii)  Each then updates their credence function on this worldly evidence they’ve obtained. To do this, each member uses the same updating rule, either the Bayesian rule or the explanationist rule with a given boost.^18^
- Step (iii)  Each then learns the updated credence functions of the others in the group. This produces their social evidence for this round.
- Step (iv)  They then update their own credence function on this social evidence by taking the arithmetic average of their credence function and the other credence functions in the group that lie within a certain distance of theirs. The set of credence functions that lie within a certain distance of your own, Douven and Wenmackers call your *bounded confidence interval*.

They then repeat this cycle a number of times, and each time an individual begins with the credence function they reached at the end of the previous cycle.

Douven and Wenmackers use simulation techniques to see how this group of individuals perform for different updating rules used in step (ii) and different specifications of how close a credence function must lie to yours in order to be included in your bounded confidence interval and thus in the average in step (iv). The updating rules they consider in step (ii) are the explanationist’s rule for different values of *c*, the reward that the rule distributes equally among the best explanations of the evidence. That is, for $c = 0$, this update rule is just the Bayesian rule, while for $c > 0$, it gives a little boost to whichever hypothesis best explains the evidence *E*, where providing the best explanation for a series of coin tosses amounts to making it most likely, and if two hypotheses make the evidence most likely, they split the boost between them. Douven and Wenmackers consider $c = 0, 0.1, \ldots , 0.9, 1$. For each rule, specified by *c*, they also consider different sizes of bounded confidence intervals. These are specified by the parameter $\delta$. Your bounded confidence interval for $\delta$ includes each credence function for which the average difference between the credences it assigns and the credences you assign is at most $\delta$. Thus, $\delta = 0$ is the most exclusive, and includes only your own credence function, while $\delta = 1$ is the most inclusive, and includes all credence functions in the group. Again, Douven and Wenmackers consider $\delta = 0, 0.1, \ldots , 0.9, 1$. Here are two of their main results:

(i) For each hypothesis other than $p = 0.1$ or 0.9, there is an explanationist rule and bounded confidence interval (i.e. $c > 0$ and some specific $\delta$) that gives rise to a lower average inaccuracy at the end of the process than the Bayesian rule with any bounded confidence interval (i.e. $c = 0$ and any $\delta$).
(ii) There is an averaging explanationist rule and bounded confidence interval (i.e. $c > 0$ and $\delta > 0$) such that, for each hypothesis other than $p = 0, 0.1, 0.9, 1$, it gives rise to lower average inaccuracy than the Bayesian rule with any bounded confidence interval (i.e. $c = 0$ and any $\delta$).

Inaccuracy is measured by the Brier score throughout.

Now, you can ask whether these results are enough to tell so strongly in favour of explanationism, but that isn’t my concern here. Rather, I want to focus on a more fundamental problem: Douven and Wenmackers’ argument doesn’t really compare Bayes’ Rule with explanationism. Instead, it compares Bayesian-rule-for-worldly-data-plus-Averaging-for-social-data with Explanationist-rule-for-worldly-data-plus-Averaging-for-social-data. So their simulation results don’t really impugn Bayesianism, because the average inaccuracies that they attribute to the Bayesian updating rule don’t arise from it. They arise from using Bayesianism in step (ii), but something quite different in step (iv). Douven and Wenmackers ask the Bayesian to respond to the social evidence they receive using a non-Bayesian rule, namely, Averaging. And Averaging lies far from the Bayesian rule.^19^

Why, then, do Douven and Wenmackers use Averaging rather than Bayes’ Rule for step (iv)? Here is their motivation:

[T]aking a convex combination of the probability functions of the individual agents in a group is the best studied method of forming social probability functions. Authors concerned with social probability functions have mostly considered assigning different weights to the probability functions of the various agents, typically in order to reflect agents’ opinions about other agents’ expertise or past performance. The averaging part of our update rule is in some regards simpler and in others less simple than those procedures. It is simpler in that we form probability functions from individual probability functions by taking only straight averages of individual probability functions, and it is less simple in that we do not take a straight average of the probability functions of all given agents, but only of those whose probability function is close enough to that of the agent whose probability is being updated. (Douven and Wenmackers 2017, p. 552)

In some sense, they’re right. Averaging or linear pooling or taking a convex combination of individual credence functions is indeed the best studied method of forming social credence functions. And there are good justifications for it: János Aczél and Carl Wagner and, independently, Kevin J. McConway, give a neat axiomatic characterization (Aczél and Wagner 1980; McConway 1981); and indeed I have argued that there are accuracy-based reasons to use it in particular cases (Pettigrew 2019). The problem is that our situation in step (iv) is not the sort of situation in which you should use Averaging. Arguments for Averaging concern those situations in which you have a group of individuals, possibly experts, and each has a credence function over the same set of propositions, and you want to produce a single credence function that could be called the group’s collective credence function. Thus, for instance, if I wish to give the SAGE group’s collective credence that there will be a safe and effective SARS-CoV-2 vaccine by March 2021, I might take the average of their individual credences. But this is quite a different task from the one that faces me as the first individual when I reach step (iv) of Douven and Wenmackers’ process. There, I already have credences in the propositions in question. What’s more, I know how the other individuals update and the sort of evidence they will have received, even if I don’t know which particular evidence of that sort they have. And that allows me to infer from their credences after the update at step (ii) a lot about the evidence they receive. And I have opinions about the propositions in question conditional on the different evidence my fellow group members received. And so, in this situation, I’m not trying to summarise our individual opinions as a single opinion. Rather, I’m trying to use their opinions as evidence to inform my own. And, in that case, the Bayesian rule is better than Averaging. So, in order to show that explanationism is superior to Bayesianism in some respect, it doesn’t help to compare Bayesianism at step (ii) + Averaging at step (iv) with explanationism at (ii) + Averaging at (iv). It would be better to compare Bayesianism at (ii) and (iv) with explanationism at (ii) and (iv).

So how do things look if we do that? Well, it turns out that we don’t need simulations to answer that question. We can simply appeal to the accuracy arguments we mentioned above: the expected accuracy argument for picking the Bayesian rule on the basis of your prior, and the accuracy dominance argument for picking a prior-rule pair where the rule is the Bayesian rule applied to the prior.

You might respond to this objection by arguing that applying the Bayesian rule at (iv) is all well and good if you are a computer or a robot, but it might require computation that is either not feasible for an ordinary person, or feasible but not worth their while. After all, it might seem to require a great deal of work to extract from those posteriors the evidence that gave rise to them and thus the evidence on which you are going to update using the Bayesian rule at (iv). Suppose each individual has drawn and replaced ten balls at (i). Then the possible evidence an individual might have received falls into eleven groups: those in which they drew and replaced no violet balls, those in which they drew and replaced one violet ball, and so on. Thus, for each of these possibilities, I would have to calculate at (iv) the posterior that they would have mandated. Only then could I compare those with the posteriors that my fellow group members have reported in order to find out what evidence they have. Surely it would be a lot easier to apply Averaging directly to the reported posteriors, even if by doing so I sacrifice some accuracy.

I agree. It would be a chore to extract that evidence. But thankfully this is not the only option. Thanks to a beautiful result due to Jean Baccelli and Rush Stewart, we can achieve the same effect by using geometric pooling instead of the linear pooling that Douven and Wenmackers use (Baccelli and Stewart ms). Given a set of credence functions $p_1, \ldots , p_n$, their straight geometric pool is defined as follows:

$$\begin{aligned} \mathrm {GP}(p_1, \ldots , p_n)(w) =\frac{ \root n \of {\prod ^n_{i=1} p_i(w)}}{\sum _{w' \in W} \root n \of {\prod ^n_{i=1} p_i(w')}} \end{aligned}$$

That is, instead of taking the arithmetic mean of the credences in each world, we take their geometric mean and normalise. We then have Baccelli and Stewart’s central result:

### Theorem 5

For any prior *p*, if $E_1, \ldots , E_m$ are compatible pieces of evidence, then

$$\begin{aligned} p\left( -|\bigcap ^n_{i=1} E_i\right) = \mathrm {GP}(p(-|E_1), \ldots , p(-|E_n)) \end{aligned}$$

Thus, I needn’t actually extract the evidence from the reported posteriors. I can simply apply an alternative pooling method at (iv). Providing all agents share the same prior, as Douven assumes, that is equivalent to applying the Bayesian rule to the extracted evidence and thus has the same advantages when assessed for accuracy.

## Choosing your intellectual trajectory

I’d like to finish by taking up a challenge that Douven lays down in passing. He writes:

[I]n science, we rarely just happen across useful data. Typically, we must actively search for data, in the many areas of science that rely on experimentation even produce our data. Because our time is limited, as is our funding, we constantly have to make decisions as to which instruments (telescopes, microscopes, etc.) to construct, which expeditions to undertake, which experiments to run, and so on. Such decisions will be informed by which hypotheses we deem most promising. Had we deemed hypothesis *H* promising, and had we wanted to compare that with the hypothesis currently dominant in our field, we might have run a different set of experiments than we actually did, given that in fact we deemed $H'$ more promising than *H* and were mainly interested in comparing $H'$ with the received doctrine. Which hypothesis or hypotheses we deem most promising, and most worthy of spending our limited resources on, will at least in part depend on how probable they appear to us, compared to their most direct rivals. If (say) a Bayesian update makes *H* more probable than $H'$, while the opposite will be the case if we update via some non-Bayesian update rule, then our decision to use one of these rules may put us on a very different research path with very different downstream consequences than if we had decided to use the other rule. Which of these paths will eventually lead us to have the more accurate representation of the world will have nothing to do with which of the rules minimizes expected inaccuracy of the piece of evidence now lying before us. (Douven 2021, p. 107)

Douven is right, of course. What credences our update rule bestows on us will determine not just how we’ll choose when faced with practical decisions, such as whether or not to publicly announce a new medical treatment, but also how we’ll choose when faced with an intellectual decision, such as which experiment to run next, which hypothesis to pursue, and so on. So, even if we focus only on our purely epistemic goal of accuracy, we’ll want credences that lead us to choose how to gather evidence in a way that maximises the accuracy we obtain after we choose them, perform them, and update on the results. But of course we have a ready-made answer to that challenge. The expected pragmatic argument for Bayes’ Rule applies just as well when the options between which you’ll choose after updating are whether to pursue one hypothesis or another, or whether to conduct this experiment or that one, and when the utilities that attach to those hypotheses at the different possible worlds are given by the accuracy of the credence functions you’ll end up with if you do pursue that intellectual trajectory.

## Conclusion

Credences play at least two roles in our lives. They guide our actions and they represent the world. When we decide how we’ll update our credences in response to evidence, we should pick a rule that leads to credences that play those roles well. Bas van Fraassen argued that there is a particular way in which updating other than as Bayes’ Rule requires leads to credences that guide action poorly. But, as Igor Douven points out, it’s a pretty weak argument. Fortunately for Bayes’ Rule, there are now much stronger arguments in its favour. One focusses on the pragmatic value of credences, while two further arguments appeal to their epistemic value. Together, they allow us to answer the sorts of objections to Bayes’ Rule that Igor Douven has raised using simulations of the two ways of updating.

This is good news for Bayesians. Is it bad news for those who think that inference to the best explanation is an important and correct rule of inference? I think not. If I have convinced you of anything at all, it can only be that any inference to the best explanation should not require a boost to hypotheses beyond what can be incorporated into a prior credence function and what Bayes’ Rule already gives them. But leaves a lot of room for inference to the best explanation to play a role within the confines of Probabilism and Bayes’ Rule.

## Footnotes

1 Though see Lange (2020) for a more nuanced understanding of how the explanatory quality of hypotheses (or ‘loveliness’, as it has come to be called) relates to our posterior credences in them.

2 One of the nice features of Bayes’ Rule is that you get the same result if you update by conditioning on your total evidence or just on your new evidence. After all, if $q(X) = p(X|E)$, then $q(X|F) = p(X|EF)$. But this is not true for the explanationist’s rival updating rules, which we’ll describe below. So it will be easier to state all candidate updating rules as operating on total evidence.

3 For more detailed accounts that fit inference to the best explanation inside Bayesianism, see Okasha (2000), McGrew (2003), Lipton (2004), Huemer (2009), Henderson (2014), Cabrera (2017), Schupbach (2018) and Dellsén (2020). For an argument that it cannot fit even with Probabilism let alone Bayes’ Rule, see Climenhaga (2017).

4 Recall: $\beta$ is Bayesian for *p* if, whenever $p(E) > 0$,

$$\begin{aligned} p^\beta _E(H_i) = \frac{p(E|H_i)p(H_i)}{\sum ^n_{j=1} p(E|H_j)p(H_j)} \end{aligned}$$

5 Standard formulations of inference to the best explanation assume that there is just one best explanation—that is, there is never a tie for the top spot. Douven, in contrast, formulates the claim a little more generally. He allows that there might be situations in which two or more hypotheses provide equally good explanations that are better than the explanations that all the competing hypotheses provide. We follow him in doing that.

6 We can now see why it is important to specify that update rules go to work on the prior and the *total* evidence and not just the *new* evidence. In our urn example, suppose you first draw a violet ball and replace it; you update using an explanationist rule $\varepsilon$; next, you draw a green ball and replace it; you update again using an explanationist rule $\varepsilon$. For the first update, your new evidence and total evidence are the same—the first ball drawn is violet—and both are best explained by $H_1$, so that gets the boost. For your second update, your new evidence is that the second draw was green: this is best explained by $H_2$; so that would then get the boost all to itself. But your total evidence is that the first draw was violet and the second was green: this is equally well explained by both hypotheses; so they would share the boost equally between themselves. So we get two different rules depending on whether they act on the new evidence or the total evidence. That distinguishes the explanationist approach from the Bayesian one. The explanationist rules that Douven consider are the one that acts on the total evidence, and those are the versions I’ll consider throughout.

7 Briggs (2009) gives a particularly clear presentation of the argument.

8 It suffices to show that

$$\begin{aligned} \sum ^n_{i=1} \sum ^m_{j=1} q(H_iE_j) = \sum ^n_{i=1} \sum ^m_{j=1}p^\alpha _{E_j} (H_i)q(E_j) = \sum ^m_{j=1} q(E_j) \sum ^n_{i=1} p^\alpha _{E_j}(H_i) = \sum ^m_{j=1} q(E_j) = 1 \end{aligned}$$

9 Thanks to an anonymous referee for encouraging me to clarify this.

10 It’s pretty clear that Savage already knew Good’s theorem when he wrote *The Foundations of Statistics* (Savage 1954, Section 7.3).

11 We assume here that your evidence is factive.

12 We assume that total evidence is cumulative, so that your total evidence at a later time is at least as strong as your total evidence at an earlier time. It therefore follows that each ${\mathcal {E}}_{i+1}$ is a fine-graining of ${\mathcal {E}}_i$. That is, for each proposition in ${\mathcal {E}}_i$, there is a set of propositions in ${\mathcal {E}}_{i+1}$ that partitions it.

13 Here’s how to do that: Suppose decision problem *D* consists of available acts *A* and $D'$ consists of available acts $A'$. Then define $D \times D'$ to be the decision problem with available acts $A \times A' = \{(a, a') : a \in A \ \& \ a' \in A'\}$, where $(a, a')(w) = a(w) + a'(w)$.

14 All page and footnote references to Douven (2021) are from the manuscript version.

15 For arguments in their favour, see Oddie (1997), Greaves and Wallace (2006), Joyce (2009) and Pettigrew (2016a).

16 This answers Douven’s objection in footnote 74 (Douven 2021).

17 Since the uniform prior is regular, there is just one Bayesian update rule for it and one explanationist update rule.

18 Again, since we assume each individual has the uniform prior over the hypotheses, and since the uniform prior is regular, there is just one Bayesian rule and one explanationist rule for each boost.

19 For more on the tension between Averaging and the Bayesian rule, see Dawid et al. (1995), Bradley (2018) and Dawid and Mortera (2020).

## References

[CR1] Aczél J, Wagner CG (1980). A characterization of weighted arithmetic means. SIAM Journal on Algebraic Discrete Methods.

[CR2] Ahlstrom-Vij, K., & Dunn, J. (Eds.). (2018). *Epistemic consequentialism*. Oxford University Press.

[CR3] Allais M (1953). Le comportement de l homme rationnel devant le risque: critique des postulats et axiomes de lécole Américaine. Econometrica.

[CR4] Baccelli, J., & Stewart, R. (ms). *Support for geometric pooling*. Unpublished manuscript.

[CR5] Bradley R (2018). Learning from others: Conditioning versus averaging. Theory and Decision.

[CR6] Briggs RA (2009). Distorted reflection. Philosophical Review.

[CR7] Briggs RA, Pettigrew R (2020). An accuracy-dominance argument for conditionalization. Noûs.

[CR8] Brown PM (1976). Conditionalization and expected utility. Philosophy of Science.

[CR9] Buchak, L. (2013). *Risk and rationality*. Oxford University Press.

[CR10] Cabrera F (2017). Can there be a Bayesian explanationism? On the prospects of a productive partnership. Synthese.

[CR11] Climenhaga N (2017). Inference to the best explanation made incoherent. The Journal of Philosophy.

[CR12] Dawid P, DeGroot MH, Mortera J (1995). Coherent combination of experts opinions. Test.

[CR13] Dawid P, Mortera J (2020). Resolving some contradictions in the theory of linear opinion pools. Theory and Decision.

[CR14] de Finetti, B. (1937 [1980]). Foresight: Its logical laws, its subjective sources. In H. E. Kyburg, & H. E. K. Smokler (Eds.) *Studies in subjective probability*. Robert E. Kreiger Publishing Co.

[CR15] Dellsén F (2020). The heuristic conception of inference to the best explanation. Philosophical Studies.

[CR16] Douven I (1999). Inference to the best explanation made coherent. Philosophy of Science (Proceedings).

[CR17] Douven I (2013). Inference to the best explanation, Dutch books, and inaccuracy minimization. Philosophical Quarterly.

[CR18] Douven, I. (2017). Abduction. In E. N. Zalta (Ed.) *The Stanford encyclopedia of philosophy*. Metaphysics Research Lab, Stanford University, Summer 2017 ed.

[CR19] Douven, I. (2021). *The art of abduction*. MIT Press.

[CR20] Douven I, Wenmackers S (2017). Inference to the best explanation versus Bayess rule in a social setting. British Journal for the Philosophy of Science.

[CR21] Elstein, D. Y., & Jenkins, C. S. I. (2020). The truth fairy and the indirect epistemic consequentialist. In P. Graham & N. J. L. L. Pedersen (Eds.), Epistemic entitlement (pp. 344–360). Oxford University Press.

[CR22] Good IJ (1967). On the principle of total evidence. The British Journal for the Philosophy of Science.

[CR23] Greaves H (2013). Epistemic decision theory. Mind.

[CR24] Greaves H, Wallace D (2006). Justifying conditionalization: Conditionalization maximizes expected epistemic utility. Mind.

[CR25] Henderson L (2014). Bayesianism and inference to the best explanation. British Journal for the Philosophy of Science.

[CR26] Horowitz S (2014). Immoderately rational. Philosophical Studies.

[CR27] Huemer M (2009). Explanationist aid for the theory of inductive logic. British Journal for the Philosophy of Science.

[CR28] Jenkins CS (2007). Entitlement and rationality. Synthese.

[CR29] Joyce JM (1998). A nonpragmatic vindication of probabilism. Philosophy of Science.

[CR30] Joyce, J. M. (2009). Accuracy and coherence: Prospects for an alethic epistemology of partial belief. In F. Huber & C. Schmidt-Petri (Eds.), *Degrees of belief.* Springer.

[CR31] Lange, M. (2020). Putting explanation back into inference to the best explanation. *Noûs*. 10.1111/nous.12349.

[CR32] Leitgeb H, Pettigrew R (2010). An objective justification of Bayesianism II: The consequences of minimizing inaccuracy. Philosophy of Science.

[CR33] Levinstein BA (2015). With all due respect: The macro-epistemology of disagreement. Philosophers Imprint.

[CR34] Lewis, D. (1980). A Subjectivists guide to objective chance. In R. C. Jeffrey (Ed.), *Studies in inductive logic and probability.* (Vol. II). University of California Press.

[CR35] Lewis, D. (1999). Why conditionalize? *Papers in metaphysics and epistemology* (pp. 403–407). Cambridge University Press.

[CR36] Lipton, P. (2004). *Inference to the best explanation* (2nd ed.). Routledge.

[CR37] McConway KJ (1981). Marginalization and linear opinion pools. Journal of the American Statistical Association.

[CR38] McGrew T (2003). Confirmation, heuristics, and explanatory reasoning. British Journal for the Philosophy of Science.

[CR39] Nielsen, M. (2021). Accuracy-dominance and conditionalization. *Philosophical Studies*. 10.1007/s11098-020-01598-6.

[CR40] Oddie G (1997). Conditionalization, cogency, and cognitive value. British Journal for the Philosophy of Science.

[CR41] Okasha S (2000). Van Fraassens critique of inference to the best explanation. Studies in the History and Philosophy of Science.

[CR42] Pettigrew R (2013). A new epistemic utility argument for the principal principle. Episteme.

[CR43] Pettigrew, R. (2016a). *Accuracy and the laws of credence*. Oxford University Press.

[CR44] Pettigrew R (2016). Accuracy, risk, and the principle of indifference. Philosophy and Phenomenological Research.

[CR45] Pettigrew, R. (2019). On the accuracy of group credences. In T. S. Gendler & J. Hawthorne (Eds.), *Oxford studies in epistemology. * (Vol. 6). Oxford University Press.

[CR46] Pettigrew, R. (2020). *The Dutch book argument. Elements in decision theory and philosophy*. Cambridge University Press.

[CR47] Quiggin, J. (1993). *Generalized expected utility theory: The rank-dependent model*. Kluwer Academic Publishers.

[CR48] Ramsey, F. P. (1926 [1931]). Truth and probability. In R. B. Braithwaite (Ed.) The foundations of mathematics and other logical essays, Chap. VII (pp. 156–198). Kegan, Paul, Trench, Trubner & Co.

[CR49] Savage, L. J. (1954). *The foundations of statistics*. Wiley.

[CR50] Schoenfield M (2016). An accuracy-based approach to higher-order evidence. Philosophy and Phenomenological Research.

[CR51] Schoenfield M (2019). Permissivism and the value of rationality: A challenge to the uniqueness thesis. Philosophy and Phenomenological Research.

[CR52] Schupbach, J. N. (2018). Inference to the best explanation, cleaned up and made respectable. In K. McCain & T. Poston (Eds.), *Best explanations: New essays on inference to the best explanation*. Oxford University Press.

[CR53] van Fraassen, B. C. (1989). *Laws and symmetry*. Oxford University Press.

[CR54] Weisberg J (2009). Locating IBE in the Bayesian framework. Synthese.

